# COVID-19 Pandemic Psychological Impact and Volunteering Experience Perceptions of Medical Students after 2 Years

**DOI:** 10.3390/ijerph19127532

**Published:** 2022-06-20

**Authors:** Esperanza L. Gómez-Durán, Carles Martin Fumadó, Aina M. Gassó, Sandra Díaz, Andrea Miranda-Mendizabal, Carlos G. Forero, Montserrat Virumbrales

**Affiliations:** 1 School of Medicine, Universitat Internacional de Catalunya, 08017 Barcelona, Spain; elgomez@uic.es (E.L.G.-D.); ainagasso@uic.es (A.M.G.); sdiaz@uic.es (S.D.); aemiranda@uic.es (A.M.-M.); cgarciaf@uic.es (C.G.F.); montsev@uic.es (M.V.); 2 Integral Care Program for Sick Health Professionals, Galatea Clinic, Galatea Foundation, 08017 Barcelona, Spain; 3 Professional Liability and Legal Medicine, Universitat Autònoma de Barcelona, 08017 Barcelona, Spain

**Keywords:** COVID-19, volunteering, psychological impact, physicians

## Abstract

Undergraduate healthcare students were mobilized to support healthcare systems during the COVID-19 pandemic, but we have scarce information regarding their experience and its impact on their wellbeing. An anonymous online survey was conducted among undergraduate students and recently graduated physicians of a medical university in Spain, regarding their symptoms and volunteering experience during the initial months of the Spanish COVID-19 pandemic. Respondents showed a high prevalence of perceived stress, anxiety, and depressive symptoms, measured by the PHQ-9 and GAD-7. 14.5% reported healthcare-related volunteering tasks. Volunteering was a satisfactory experience for most of the respondents and the majority felt ready to do volunteering tasks (66.6%). Yet, 16.6% acknowledged not getting appropriate specific-task education before starting, 20.8% reported not having appropriate supervision, and 33.3% feel they did not have proper protective equipment. More than half of volunteers feared getting infected, more than 70% feared infecting their relatives or friends, and 54.2% reported stigmatization. Volunteers showed significantly higher stress, anxiety, and depression scores than the rest of the respondents, and 32% reported a highly traumatic event during volunteering, with high scores on the IES-R in the 16% of volunteers. Our results should help guide future potential volunteering processes in emergencies, enhance academic programs at medical schools and provide valuable data for psychological support services.

## 1. Introduction

During the COVID-19 pandemic, a rise in psychological symptoms such as anxiety, perceived stress, and depression was recorded in the general population [1]. University students usually present high rates of mental health disorders [2,3,4,5,6] and medical students are recognized as an at-risk subgroup, with significantly larger rates than the general population, even under normal circumstances. Besides being less likely to seek support when affected by psychological distress, their distinctive personality traits, together with their deeper understanding of COVID-19 severity, might have made them especially sensitive to the pandemic-related distress [7,8].

Furthermore, many medical students actively engaged in the fight against COVID-19. In-person academic activities were suspended at the beginning of the pandemic in 2020 and students were sent home to continue their studies remotely through virtual learning systems. The COVID-19 pandemic increased staffing needs in healthcare systems. Healthcare undergraduate students, as critical members of human capital in the health sector, were mobilized to support healthcare systems during the emergency. They were of real assistance during the toughest times of the pandemic and have contributed to the health system’s resilience [9,10].

In Spain, healthcare students’ involvement in the COVID-19 pandemic response depended on their willingness to volunteer and readiness to practice. They showed specialized knowledge and skills that were most useful in the fight against the pandemic but, little is known about their feelings and readiness to take action. Mühlbauer et al., examined the influence of psychological factors on students’ decisions to respond to volunteering calls [11]. Students’ decisions to volunteer revealed both altruistic and introjected motivations since a sense of duty and desire to help were the most important reasons for volunteering. Depressive symptoms and lack of time made volunteering less likely, yet, resilience and COVID-19 related anxieties did not seem to have had any influence on the decision to volunteer or not [11]. 

A previous study of Spanish medical students explored, through a phenomenological qualitative approach, the perceptions of students for voluntary incorporation into the healthcare system. Out of the global sample, 85% of students reported having voluntarily joined the system for ethical and moral reasons, and the main reported feelings regarding their mood were negative, represented by uncertainty, nervousness, and fear [10].

During the pandemic, medical students received concrete recommendations when volunteering to work in healthcare: they were to undergo appropriate training, not undertake any activity beyond their level of competence, and receive continuous supervision and adequate personal protective equipment [12]. Yet, little is known about their actual level of confidence with the work performed, their actual tasks, and work and safety conditions. Fear of contagion has been reported in volunteer medical students. For instance, Khalid et al., reported that the medical students they surveyed strongly agreed that they would have been willing to volunteer during the COVID-19 pandemic if they had been provided personal protective equipment (49.0%) and if their parents had been supportive of their decision to volunteer (44.5%). Medical students (40.5%) felt that they were somewhat likely to get infected while volunteering during the COVID-19 pandemic and 55.5% felt that in turn, they were extremely likely to infect their families as well. In the event of COVID-19 infection, 42% of medical students felt that they would recover without hospitalization [13]. Mihatsch et al., and Zhang et al., also reported that students were more concerned about infecting other patients and relatives than themselves [14,15].

Positive aspects of volunteering should also be highlighted. It has been suggested that volunteering during the pandemic has provided an active learning environment, with high levels of skills development and widened awareness, that may compensate for the pandemic’ impact on medical education [16]. Other aspects, such as the valuable experience in interprofessional collaboration have also been cited, as students were integrated into established healthcare teams [17].

Furthermore, the vast majority of medical students said they would be willing to work as medical assistants again and that this experience would not affect their career choice [15]. Additionally, a study carried out by Office [18] (2020) showed that healthcare student volunteers may have felt that their volunteering experience was impactful for both themselves and the patients.

Yet, volunteering during the COVID-19 pandemic made medical students potentially subject to the same psychological consequences as other healthcare workers on the frontline, albeit with less professional experience on which to rely [19]. In this regard, the Depression, Anxiety, and Stress Scale (DASS)-21 has been previously used to survey the psychological burden of volunteer students, and Zhang et al., showed that 26.8% of the medical students reported depression, 20.2% reported anxiety and 11.1% reported perceived stress [15]. The volunteers’ negative emotions were more pronounced before work and diminished gradually, while positive emotions emerged.

Despite all these worldwide studies, there is scarce evidence regarding the Spanish experience and none of the cited studies performed a comprehensive assessment, neither did they evaluate posttraumatic or positive experience impact specifically. Little is known about the impact of the pandemic situation on the psychological well-being of students who volunteered as assistants in comparison to their peers. Such a study would provide valuable insights about risk factors specifically associated with healthcare provision during the COVID-19 pandemic, as well as their impact on medical students. In this study, we aimed to assess the impact of the pandemic on the psychological well-being of medical students at a Spanish university (Universitat Internacional de Catalunya—UIC, Sant Cugat del Vallés, Spain). Specifically, we compared students who voluntarily served as healthcare collaborators in healthcare units with those who did not collaborate with healthcare centers during the first six months of the COVID-19 pandemic (from March to August 2020).

## 2. Materials and Methods

### 2.1. Sample and Data Collection Procedures

A cross-sectional study on undergraduate and recently graduated medical students at UIC was conducted between December 2021 and March 2022. All students above third grade and recently graduated (egressed from 6th course) during 2020 were invited to participate. The survey was distributed to approximately 600 students through the institutional email system. Links to the survey were sent individually and were encrypted. Data was anonymous with no personal identification data that might allow recognizing participants’ identities.

The UIC Ethics in Research Committee approved the protocol (MED-2021-10), and participants provided informed consent before beginning the survey. There was no penalty for withdrawing and no compensation was associated with the participation.

### 2.2. Variables

The survey was composed of the sections detailed below. It was designed purposefully by the researchers. The following variables were gathered: socio-demographics (age and gender), social interactions during the pandemic, year of medical studies, COVID infection status and symptomatology (infections status, severe COVID-19), the infection status of relatives, and severe illness of relatives and close ones. Also, we gathered academic information, willingness to act as healthcare volunteers, readiness to practice, volunteering activities and conditions, and students’ overall opinions regarding the volunteering experience. For this study, healthcare volunteering was conceptualized as providing services, assistance, or support at a healthcare facility in response to the COVID-19 outbreak.

Finally, we measured students’ psychological status using self-reported questionnaires. Spanish adapted versions of the following instruments were used after reviewing the items’ wording to refer to the initial months of the pandemic.

-The Patient Health Questionnaire-9 (PHQ-9) [20] is a self-administered scale consisting of 9 items, rated on a Likert scale ranging from 0 (not at all) to 3 (almost every day). No substantial differences in the pooled sensitivity and specificity for a range of cut-off scores (8–11) have been reported. We reported results for both cut-off scores of 8 and 11. The Spanish version has shown comparable reliability and validity evidences to those of the original version [21].

-Generalized Anxiety Disorder-7 (GAD-7) is also a self-administered scale to assess the severity of anxiety disorders. It consists of 7 items directly aimed at the measurement of anxiety symptomatology. Each item was rated on a Likert scale ranging from 0 (not at all) to 3 (almost every day). GAD-7 ratings reflect 4 levels of severity of anxiety disorder: none (0–4), mild (5–9), moderate (10–14), and severe (15–21). The Spanish version has shown good reliability and validity evidence, and a cut-off point for diagnoses has been established [22].

-Perceived Stress Scale (PSS) [23]. The scale is widely used as a self-report instrument used to assess the level of perceived stress and the degree to which an individual would find their life unpredictable, uncontrollable, or overwhelming. The PSS consists of 14 items with a Likert-type response format having five response options that are punctuated from 0 (“never”) to 4 (“very often”). The range of scores on the scale is from 0 to 56, with higher scores corresponding to higher levels of perceived stress [23]. The Spanish version by Remor and Carrobles has shown good levels of reliability and validity [23].

-Impact of Event Scale-Revised (IES-R) [24]. Finally, those who reported experiencing a traumatic event during COVID-19 volunteering responded to the Impact of IES-R related to the traumatic event. The IES-R is also a self-reporting instrument. It consists of 22 items and each item is rated on a Likert scale ranging from 0 (not at all) to 4 (extremely). IES-R scores reflect 4 levels of distress severity: none (0–8), mild (9–25), moderate (26–43), and severe (44–88). The IES-R was only asked to respond by students who had participated as volunteers so that we could explore how many of them reported a traumatic event during their volunteering experience.

### 2.3. Data Analysis

Sample description in sociodemographic characteristics using as percentage respondents. For the description of volunteering experiences, we computed response percentages and their 95% confidence intervals. As for psychological well-being, the prevalence of probable depression according to PHQ-9 was reported according to cut-off points established between scores of 8 and 11. Also, the severity of symptoms was estimated for PHQ-9, GAD-7, and PSS as scores, providing their means and standard errors. As for the second objective, we compared PHQ-9 probable cases of depression according to standard cut-offs in volunteers and non-volunteers using the Chi-square test. Psychological wellbeing was assessed by comparing volunteers with non-volunteers in PHQ-9, GAD-7, and PSS scores using independent sample *t*-tests; homoscedasticity assumption for *t*-test was tested using Levene’s test for equality of variances and if significant, the Welch’s correction was applied to ensure correct *p*-values. We used nominal alpha level α = 0.05 for decisions. Data was analyzed using IBM SPSS v26 (Chicago, IL, USA).

## 3. Results

The obtained response rate was 29%, with 175 students agreeing to participate in the study. Two participants were excluded since survey responses only contained gender and age. The final sample characteristics are described in Table 1.

In the sample, 78% were male, the average age was 22.45 (median 22; SD 2.344). This distribution is not significantly different regarding gender and age in the *n* = 600 sample of invited participants. Most participants (87.3%) lived with their parents. Respondents were mostly from the first five years of medical school (90.3%). About 6% of the sample was COVID-19 positive, and one participant had severe COVID-19 requiring hospital admission. COVID prevalence in the sample was 5.8% (CI95% 4.0 to 7.6%). About one-fifth (18.5%, CI95% 15.6 to 21.4%) of the participants declared to have lost or had a close person seriously ill during the pandemic.

Out of the total sample, 14.5% of participants (*n* = 25) reported participating in healthcare-related volunteering tasks from March to August 2020. Most of them did volunteering tasks for more than one month from March to August (70.8%, *n* = 17 out of 24). Students participated in a variety of tasks. Out of the 25 volunteers, twenty participants (80%, CI95% 72 to 88%) worked at the telephone service for patients; ten (40% CI95% 30.2 to 49.9%) provided clinical care, three (12% CI95% 18 to 42%) gave information to families or facilitated contact between relatives and patients, and three helped with childcare for frontline physicians and one did epidemiological tasks.

Most respondents felt they were ready to do these tasks before they volunteered (64.6%, CI95% 54.4 to 73.6%); 16% (CI95% 8.66 to 23.33%) reported not having appropriate specific-task education before starting their volunteering (16.6% out of 24), 20% (CI95% 12 to 28%) reported not having appropriate supervision during their tasks. Most respondents feared getting infected by COVID-19 before they volunteered (56.0%, CI95 46.1 to 65.9%); 48% (CI95% 38.0 to 57.9%) feared getting infected during their volunteering tasks. Furthermore, 76.0% (CI95% 67.5 to 84.54%) feared infecting their relatives or friends and 68.0% (CI95% 58.7 to 77.3%) still feared infecting someone during their volunteering experience. Overall, 32% of respondents (CI95% 22.7 to 41.3%) felt they did not have proper protective equipment during their volunteering tasks. Of the, 54.2% (CI95% 42.0 to 62.0%) felt stigmatized because of their healthcare volunteering during the first months of the pandemic, although 64% (CI95% 54.4 to 73.6%) felt positive feedback from relatives, friends, and patients.

Volunteers felt mostly satisfied by the experience (76%, CI95% 67.5 to 84.5%), and considered volunteering as a positive educative experience (72%, CI95% 63.0 to 81%). About half of them considered the experience to have a positive impact on their future career (52%, CI95% 42 to 62%) and at a personal level (CI95% 63 to 81%). Most of them considered that the interprofessional component of integration in a multidisciplinary team and collaboration with other professional profiles was very positive (76%, CI95% 67.5 to 84.5%).

The PHQ-9 average score among respondents was 7.78 (median 7.00; SD 5.955). The GAD-7 average score was 7.65 (median 7.00; SD 5.606). The PSS average score was 20.00 (median 20.00; SD 3.338). Differences regarding depressive, anxiety, and stress symptoms between those who volunteered and those who did not, are shown in Table 2.

We found significant differences in depressive symptomatology between volunteers and students who did not volunteer. Volunteers showed significantly higher levels of severity regarding depressive symptoms (11.52 vs. 7.14, *p* = 0.01), and also a higher proportion of them was above the cut-off (score ≥11) for probable depression (56.0% vs. 21.6% *p* = 0.001). Similarly, volunteers showed higher levels of anxiety (10.68 vs. 7.14, *p* = 0.012) and stress (21.68 vs. 19.72) than their non-volunteer peers. PHQ-9, GAD-7, and PSS Severity scores were not heteroscedastic according to Levenes’ test.

Only eight participants reported a highly stressful or traumatic event during volunteering (32%), and the IES-R average score among them was 37.50 (median 38.50; SD 22.519). However, all of them were volunteers and, thus it was not possible to make comparisons with other students. Levels of distress severity were high for 4 of them, moderate for one participant, and mild for 2 of them; one participant that reported a highly stressful or traumatic event did not show distress according to the IES-R.

## 4. Discussion

Those who were medical students during the first months of the COVID-19 pandemic are already or will soon become frontline healthcare workers, but little is known about how they experienced those tough days. Earlier studies focused on medical students’ attitudes, motivations, and willingness to volunteer during the COVID-19 outbreak, or the role of volunteering in the education of future healthcare professionals and the formation of their professional identity, but it has been highlighted that there is still a great shortage of research on the student’s experiences during the pandemic [25]. 

We found a substantial level of perceived stress in our sample, as measured by the PSS Medical students show a high prevalence of perceived stress and the COVID-19 pandemic, added to the academic workload and responsibilities students bear as future health professionals, became another stress factor in undergraduate medical training. Perceived stress during the COVID-19 pandemic has been previously reported as high among medical students. Duarte et al., performed a transversal assessment of students attending a medical school in northern Portugal through an online survey, and PSS scored an average of 21, with a standard deviation of 7 [26].

In addition to the level of perceived stress, studies have reported increased levels of anxiety and depression during the pandemic. Healthcare worker-first responders in a sample from the United States reported a GAD-7 mean score of 12.55 and a PHQ-9 mean score of 16.57, which were the highest mean scores among all groups that Guerrini et al., considered [27]. Likewise, Saraswathi et al., demonstrated a significant increase in the prevalence of depression (33.2%) and anxiety (21.2%) during the COVID-19 pandemic in medical students [28].

Our results regarding anxiety scores were similar to those previously reported in the United States, with a GAD-7 average score of 7.65 (SD 5.606). Guo et al., reported a GAD-7 score for undergraduate medical students of 7.47 (SD 5.27) during the June-August 2020 period in the United States [29]. Halperin et al., performed another cross-sectional study in the United States and reported a mean GAD-7 score of 7.3 (SD 2.1–12.5) [30]. Sartorao Filho et al., reported an average GAD-7 score for anxiety of 9.18 (SD 4.75) in their cross-sectional study of medical students from a medical school in Brazil, conducted in May 2020 [31]. The percent distribution of severity of anxiety symptoms in our sample was similar to that reported by Guo et al. [29]. According to the GAD-7 scores in Guo et al.’s study, 33.92% of first through fourth-year medical students did not have any symptoms of anxiety, 34.98% showed mild anxiety, 19.25% reported moderate anxiety, and 11.85% had severe anxiety [29]. According to our results, 32.4% of students did not report symptoms of anxiety, 32.9% showed mild anxiety, 23.1% had moderate anxiety, and 11.6% had severe anxiety.

Regarding depressive symptoms, our results showed an average score on PHQ-9 of 7.78 (median 7.00; SD 5.955). Sartorao Filho et al., reported an average PHQ-9 score for depression of 12.72 (median 12.72; SD 6.62) among medical students, whilst Halperin et al., reported a mean score on was 6.5 (SD 0.9–12.1) [30,31].

Factors contributing to these outcomes merit further study, but our results, conditioned by the time elapsed, confirm previously reported rates of perceived stress, anxious and depressive symptoms and underline that the imprint left by those initial months of the pandemic on the students was one of a high level and last in their memories two years later. Furthermore, 38.7% and 32.9% of the surveyed students reported currently feeling the same depressive and anxious symptoms respectively. Therefore, the long-lasting impact of the pandemic experience on medical students should be expected, and resources should remain active for a longer time before consequences can be ameliorated.

Remarkably, volunteering was a satisfactory and enriching experience for most of the respondents in our sample. In this line, medical students surveyed in Bazán et al.’s study also revealed a high level of satisfaction from volunteering activities [9]. Regarding volunteering experiences, our sample engaged in more than one task, and tasks performed varied widely. Such result is similar to those in previous studies, with telephonic patient care and COVID-19 surveillance tasks being the most frequently reported activities [9]. Arguably, the experience gained when facing the crisis by providing aid in a variety of tasks is likely to benefit volunteers in the future, and so did our respondents consider. Hughes et al., even suggested this active learning environment may compensate for the pandemic’s impact on medical education [16]. Our student’s positive opinion about integration into healthcare teams is in line with results by Buckland claiming that pandemic volunteering is a valuable interprofessional collaborative experience [17].

However, the positive volunteering experience carried out during the pandemic was overshadowed by the conditions and circumstances in which this experience took place. Between one and two out of 10 students who volunteered reported insufficient orientation and supervision for the tasks they performed. Our results concur with those of Domaradzki’s qualitative study, which underpinned that many students felt unprepared for dealing with the pandemic, and for others, volunteering during COVID-19 was a source of serious burden [25]. Perceived insufficient medical knowledge and skills have been pointed out as a major concern regarding volunteering [32]. In a previous Spanish survey study with nursing and medical students, it was also reported that 65.3% of students did not feel prepared or felt they were barely prepared to attend COVID-19 cases and only 18.6% of the students had received some kind of specific training on COVID-19 [33]. Yet, the majority of our sample was lower than the fourth year at the time of the COVID-19 Spanish outbreak (59.6%) which surely influenced their self-assessment of their level of readiness to perform professional activities.

Moreover, volunteers in our sample felt fear of infection, and many perceived that they did not have sufficient protection during their activities. Our societies and healthcare systems were not ready for the COVID-19 pandemic, and healthcare was provided under unconventional situations and inadequate occupational health conditions were frequent, under the general catastrophic scenario [34]. Concerns about becoming infected and endangering loved ones have repeatedly been reported by healthcare workers during pandemics [35]. In our sample, more than half of volunteering students feared getting infected before and during their volunteering tasks, and more than 70% feared infecting their relatives or friends. Mihatsch et al., and Zhang et al., also reported that students were more concerned about infecting other patients and relatives than themselves [14,15].

In our sample, most respondents feared getting infected by COVID-19 before they volunteered (56.0%), but the percentage dropped to 48% once they were on task. Zhang et al., reported that the volunteer’s negative emotions were more pronounced before work and diminished gradually, while positive emotions emerged [15]. The majority of students (79.3%) in Bazan et al., study admitted to some level of fear at the beginning of volunteering [9]. Yet, the majority of students surveyed by Bazan et al., reported that their level of fear decreased throughout volunteering or remained unchanged [9]. The volunteers in Bazán et al.’s study mostly feared the transmission of the SARS-CoV-2 to relatives (61.2%), contraction of the virus (24.5%), failure to fulfill entrusted duties (26.0%), and to a lesser extent, the reaction of the environment to their participation in volunteering (11.4%) [9].

Governments and healthcare systems should learn from this kind of experience and be prepared to respond to these outbreaks by adopting policies that guarantee necessary human and material resources and ensure citizens’ well-being by covering basic services, but in a way that the safety of healthcare workers is ensured [34]. Recruiting undergraduate students is an accessible resource for boosting health services, but a previously outlined contingency plan, with clear training and support for enrolled students is necessary for efficiently taking advantage of their competencies.

Alarmingly, over half of the volunteers in our sample reported being the target of stigmatization related to volunteering tasks. Discrimination and stigmatization have been previously reported targeting healthcare professionals during other epidemiological events [35] and have already been described during the COVID-19 pandemic regarding medical student volunteers [9]. As our survey asked about the early stages of the COVID-19 outbreak in Spain, the vivid fear of the unknown probably fueled stigma stronger than later on. Nevertheless, just like respondents in Domaradzki’s qualitative study [25] participants in our survey emphasized that their involvement in voluntary service during the pandemic had received positive feedback. Such feedback is known to beneficially promote the reinforcement of the decision to volunteer under potentially stressful conditions [36].

The positive perception about volunteering does not come without a personal cost. Volunteer confidence was probably more related to their capacity to perform the volunteering tasks than to their capacity to overcome and manage the psychological impact of the experience. Our volunteers showed higher depressive and anxiety symptoms than their peers. Moreover, a significantly higher proportion of respondents reached the cut-off for being considered a potential case of clinical depression. Zhang et al., reported a detection rate of depression of 26.8%, according to the Depression, Anxiety, and Stress Scale (DASS-21) scoring [15]. Zhang et al.’s study is the only previous one that reports depressive measures specifically in medical student volunteers [15]. Our figures were higher, although our students felt prepared to perform their volunteering tasks at a higher rate than previously reported [33]. However, the PHQ-9 has demonstrated excellent detection accuracy, while the DASS-21 has shown serious reasons for concern regarding its psychometric properties [37]. 

Beyond depressive symptomatology, 32% of our volunteers reported a highly stressful or traumatic event during volunteering, and the IES-R average score amongst them was 37.50 (SD 22.519). Miller et al., (2020) [38] already alerted about a high risk of post-traumatic stress among volunteering students and advocated for students’ right from a modern conception of medical education, far away from considering students as a simple workforce in hospitals. Only one out of the eight participants that reported a highly traumatic event did not show distress according to the IES-R, which would mean that 28% of those who volunteered did show posttraumatic distress. Our results are consistent with data on healthcare workers that report that 25% of them have a positive screen indicating an increased risk for posttraumatic stress disorder [19,39]. Volunteering during the COVID-19 pandemic made medical students potentially subject to the same consequences as other healthcare workers on the frontline, albeit with less professional experience on which to rely [19]. Despite levels of COVID-19 exposure in the clinical setting and, therefore, levels of acute stress, which were surely lower in students than in healthcare workers on the frontline, their lack of experience and training probably increased the traumatic potential of the experience, showing similar rates of post-traumatic symptomatology in both groups.

## 5. Limitations

There are several limitations to this study, including the small sample size. A selection bias could be expected, as the students who felt compelled to respond may not be generalizable to all medical students at the school. Our participants were recruited from one large School of Medicine in Spain, and therefore may not be representative of participants from other academic institutions, healthcare systems, or countries. Furthermore, there was an uneven distribution of respondents by years of Medical School, and the majority of our sample was lower than the fourth year at the time of the COVID-19 Spanish outbreak (59.6%). Our assessments were based only on self-reported measures, which could be impacted by information bias, and were not clinically confirmed by a medical professional, so diagnostics could not be inferred. Lastly, two years passed by after the initial months of the pandemic before we performed our study. Despite this limitation, it has been suggested that, for example, posttraumatic stress symptoms should be measured several years after the event.

On the other hand, some strengths should also be highlighted. Our study provides one of the few published reports examining stress, anxiety, and depressive symptoms in medical students, and the first to compare those who did volunteer with those who did not. To our knowledge, this is the first study to provide data on posttraumatic symptoms in medical student volunteers, so more conclusions on how medical students contributed to the response to COVID-19 will be possible as more studies in this regard emerge from various other countries.

## 6. Conclusions

Our study provides new insights into the effects of the COVID-19 pandemic on those who were medical students during the initial months of the COVID-19 pandemic. In summary, high depression, anxiety, and stress scores during those initial months are reported by medical students two years after the beginning of the COVID-19 outbreak. Furthermore, one out of three reported currently feeling the same symptoms. Symptoms were significantly higher in medical students that did healthcare volunteering during these months compared to those who did not. Approximately one out of three medical students who volunteered reported a traumatic event during their volunteering experience and one out of six suffered severe posttraumatic symptoms. Although they did not face equivalent levels of healthcare stress, our results on medical student volunteers are consistent with data on first-line healthcare workers, which underlines the potential impact of this type of situation on people with less experience and training.

Encouragingly, most volunteers considered volunteering a positive experience at an educational, professional, and personal level. Yet, according to our data, those responsible for educational institutions are expected to take this epidemic as an opportunity to improve the educational programs of future health professionals by incorporating the necessary competencies to face epidemic outbreaks in a way that helps mitigate the negative psychological impacts of the experience. Public authorities and emergency management agencies should use this valuable information for organizing the voluntary service in case of future epidemics and designing contingency plans that include training for volunteers. Support services for those who volunteered in the COVID-19 pandemic must remain active in the long term. Far beyond rejecting socially reprehensible attitudes, such as stigmatization, we all have the social responsibility to care for those who cared for us. Furthermore, understanding how medical students perceived this stressful experience is important because their mental health has implications for their future role in caring for our societies.

## Figures and Tables

**Table 1:** Sociodemographic variables of the sample.

Variables		% (*n*) [Mean, SE]
Gender	Female	78.6% (136)
	Male	20.8% (36)
	Fluid Gender	0.6% (1)
Age		[22; 0.17]
Who were you living with?	Family members	87.3% (151)
	Couple/friends/flatmates	8.7% (15)
	Alone	2.9% (5)
	Not reported	0.6% (1)
Year of Medical School at time of COVID19 outbreak	First	14.5% (25)
	Second	22.0% (38)
	Third	23.1% (40)
	Fourth	16.2% (28)
	Fifth	14.5% (25)
	Sixth	4.0% (7)
	Just graduated	1.2% (2)
	Not reported	4.6% (8)
COVID-19 infection (Yes)		5.8% (10)
Severe COVID-19 infection (Yes)		0.6% (1)
Think infected of COVID-19 to someone (Yes)		2.9% (5)
Close one seriously ill or died because of COVID-19 (Yes)		18.5% (32)
PHQ-9 score		[7.78; 0.45]
PHQ ≥ 8		57.2% (74)
PHQ ≥ 11		26.6% (46)
Still have PHQ-9 depressive symptoms	Yes	38.7% (67)
	No	50.3% (87)
	I did not have any of them	9.8% (17)
	Not answered	1.2% (2)
GAD-7		[7.65; 0.43]
	No anxiety	32.4% (56)
	Mild anxiety	32.9% (57)
	Moderate anxiety	23.1% (40)
	Severe anxiety	11.6% (20)
Still have Anxiety symptoms	Yes	32.9% (57)
	No	57.2% (99)
	I did not have any of them	9.8% (17)

**Table 2:** Differences in psychological wellbeing between volunteer participants and non-volunteer participants.

Variable	Volunteers (*n* = 25) Mean, SD [%, *n*]	Rest of the Students (*n* = 148) Mean, SD [%, *n*]	*p* ^1^
PHQ-9 score	11.52, SD 7.63	7.14, SD 5.40	0.010 ^1^
PHQ-9 ≥ 8	[60%, 15]	[39.9%, 59]	0.080 ^2^
PHQ-9 ≥ 11	[56%, 14]	[21.6%, 32]	0.001 ^2^
GAD-7 score	10.68, SD 6.22	7.14, SD 5.35	0.012 ^1^
PSS score	21.68, SD 3.06	19.72, SD 3.31	0.006 ^1^

^1^ Independent samples *t*-test, 171 degrees of freedom. ^2^ Chi-Square test, 1 degree of freedom.

## Data Availability

Not applicable.
